# Evolutionary Comparison of the Developmental/Physiological Phenotype and the Molecular Behavior of SPIRRIG Between *Arabidopsis thaliana* and *Arabis alpina*

**DOI:** 10.3389/fpls.2020.596065

**Published:** 2021-01-07

**Authors:** Lisa Stephan, Marc Jakoby, Martin Hülskamp

**Affiliations:** Botanical Institute, Biocenter, Cologne University, Cologne, Germany

**Keywords:** morphogenesis, membrane trafficking, *Arabis alpina*, BEACH domain protein, SPIRRIG, salt response

## Abstract

Beige and Chediak Higashi (BEACH) domain proteins mediate membrane-dependent processes in eukaryotic cells. The plant BEACH domain protein SPIRRIG in *A. thaliana* (AtSPI) was shown to display a similar molecular behavior as its yeast and animal homologs, along with a range of cell morphological defects. In addition, AtSPI was shown to interact with the P-body component DCP1, to differentially effect RNA levels and to be involved in the regulation of RNA stability in the context of salt stress responses. To determine, whether the dual function of SPI in apparently unrelated molecular pathways and traits is evolutionary conserved, we analyzed three *Aaspi* alleles in *Arabis alpina*. We show that the molecular behavior of the SPI protein and the role in cell morphogenesis and salt stress response are similar in the two species, though we observed distinct deviations in the phenotypic spectrum.

## Introduction

The mechanisms of cell growth and morphogenesis are in the focus of plant science for decades. Due to fully sequenced model organisms like *Arabidopsis thaliana* and excellent model systems for cell differentiation like root hairs and trichomes (Mathur and Hülskamp, 2002; Bögre et al., 2008), genetic and environmental factors regulating growth, size and shape of plant organs are better understood nowadays. Morphogenesis is thought to be controlled predominantly by endoreduplication and thereby cell size control, cytoskeletal dynamics, vesicle transport and small GTPase signaling (Guimil and Dunand, 2007).

A group of mutants with *distorted* cell shapes was identified in EMS screens in *Arabidopsis thaliana* (Koornneef et al., 1983, 1987; Hülskamp et al., 1994), which show altered trichome morphology, irregular shapes of leaf pavement cells, epidermal hypocotyl cells and epidermal root cells. The corresponding *distorted* genes were found to encode proteins of the actin regulating ARP2/3 or SCAR/WAVE complex (Mathur et al., 2003; Schwab et al., 2003; Basu et al., 2005), with one exception: the BEACH domain protein SPIRRIG (Saedler et al., 2009).

BEACH domain proteins are named after their corresponding mutations in mice and humans, the beige and Chediak Higashi (BEACH) syndromes (De Lozanne, 2003). They are known facilitators of membrane dynamics (De Lozanne, 2003) and regulators of endosomal sorting processes (Cullinane et al., 2013). In agreement with these essential functions, the BEACH domain protein family is well conserved in mammals, plants and yeast, often exhibiting several members within one species (Saedler et al., 2009).

In Arabidopsis, only the BEACH domain protein SPIRRIG has been characterized so far (Saedler et al., 2009). In the N-terminus, SPI carries tandemly repeated Armadillo domains, each approximately 42 amino acids long. Armadillo domains share a conserved three-dimensional structure of three α helices, which fold together and interact to form a surface for protein-protein interactions (Coates, 2003). The Armadillo repeats are followed by a Concanavalin A (ConA)-like lectin domain, which is thought to be involved in oligosaccharide binding and to mediate membrane fusion events (Burgess et al., 2009). In the C-terminus, SPI exhibits a Pleckstrin-Homology (PH) domain, followed by the name giving BEACH domain. PH domains were found to interact with BEACH domains to form a large groove, possibly serving as a ligand-binding site (Jogl et al., 2002). The BEACH domain of SPI is followed by several WD40 repeats, which can mediate protein-protein interactions.

The *spirrig* mutants in *A. thaliana* share all cell morphogenesis defects observed in other *distorted* mutants including lower complexity of epidermal pavement cells, weakly *distorted* and curled trichomes, disconnected and out-curled hypocotyl cells in dark grown seedlings as well as defects in vacuolar integrity (Saedler et al., 2009). AtSPI seems to act in two very different pathways. First, it was shown that AtSPI physically interacts with the ATPase Suppressor of K+-Transport Growth Defect 1 (AtSKD1) and LYST Interacting Protein 5 (AtLIP5; Steffens et al., 2017) suggesting that it functions in the context of the endosomal sorting complex required for transport (ESCRT) machinery. Second, AtSPI localizes to and facilitates the formation of processing bodies (P-bodies). In addition, *Atspi* mutations lead to pleiotropic transcriptional changes and the stabilization of several mRNAs and their recruitment to P-bodies under salt stress conditions. Also this pathway was suggested to be evolutionary conserved because the AtSPI protein interacts with DECAPPING PROTEIN 1 (DCP1) from other species (Steffens et al., 2015).

The unusual finding that the *Arabidopsis thaliana SPI* gene has a dual role in unrelated pathways and traits raises the question, whether this is specific to one species or evolutionary conserved. We therefore studied this question in another distantly related Brassicaceae species that enables us to use genetic approaches and to unambiguously identify the true *SPI* ortholog by sequence similarity and synteny. We compared the developmental/physiological and molecular function between *A. thaliana* and *Arabis alpina*. *A. alpina* is suitable for laboratory use, since it is diploid, self-fertile, has a small and mostly sequenced genome and can be transformed with *Agrobacterium tumefaciens* (Wang et al., 2009). *A. alpina* was chosen for this work, since it is sufficiently closely related to *A. thaliana* to clearly identify orthologous genes, but also distant enough (26–40 million years; Koch et al., 2006; Beilstein et al., 2010) to expect phenotypic and molecular variations. The SPI proteins of *A. thaliana* and *A. alpina* are highly conserved, especially in the C-terminus. The sequence identity reaches 94% for the whole protein and 97% for the PH-BEACH-WD40 (PBW) domain. Would *Aaspi* mutants show the same phenotypic spectrum as *Atspi* mutants? Would the molecular behavior be comparable? Using three *Aaspi* mutants identified in an exhaustive EMS screen for trichome mutants in *A. alpina* (Chopra et al., 2019) we reveal a similar molecular behavior and phenotypes, though the phenotypes show distinct differences.

## Materials and Methods

### Plant Material, Growth Conditions, and Stress Treatments

*Arabis alpina* EMS *spirrig* mutants *spi-1*, *spi-2*, and *spi-3* were confirmed by sequencing of the *SPIRRIG* (Aa_G228370) transcripts obtained from PCR (N-terminus: 5′-CGTGTTTAGAGAGAGAAAG-3′, 5′-TATGAACAACAGCAA GGTGA-3′; Internal fragment: 5′-GCAACACGAACAGAACA TT-3′, 5′-CATCGTTCAAGCTTTTTGC-3′; C-terminus: 5′-AA TCCAAAGGGTCTGAAG-3′, 5′-ACAGATGGGAGCTATACA AT-3′; GATC/Eurofins, Ebersberg). Seeds were grown on soil, or surface-sterilized and grown on full MS medium (Murashige and Skoog, 1962). The seeds were stratified for 5 days. Subsequently, plants were grown under long day conditions at 21 ± 1°C and 100 ± 20 μmol/m^2^s light intensity. To analyze plant growth in the dark, seeds were removed from light conditions after 4 h, wrapped light-tight and vertically grown for 7 days. For germination assays, seeds were grown on MS plates and MS plates supplemented with 125 mM NaCl. Over the course of 20 days, seeds were checked for breakthrough of either root or cotyledons daily under a stereo microscope. For primary root growth assays, seeds were planted on MS plates, grown vertically, and transferred to MS plates and MS plates supplemented with different concentrations of NaCl after 7 days. Growth was then documented for 4 or 8 days. Seedling growth under transpiring conditions was carried out on soil under standard conditions, plants were watered by dipping every 3–4 days with water until day 20, followed by water or water supplemented with 125 mM NaCl until day 30. For transcript measurements, two to three 5–7 day old seedlings were transferred from plates to liquid 1/2 MS or liquid 1/2 MS with 125 mM NaCl for 4 h under constant shaking and subsequently frozen with liquid nitrogen. For short term salt stress of leaves, samples were transferred to 1/2 MS with 125 mM NaCl for 60–80 min.

### Fluorescein Diacetate (FDA) Staining

Fluorescein diacetate was used as a negative stain to identify vacuole patterns in root hairs. Plants were treated with a 0.05% w/v solution of FDA (Sigma) in ddH_2_O for 15–20 min and analyzed under the fluorescence microscope.

### Agarose Imprints

Agarose imprints were used to visualize the cell shapes of epidermal pavement cells. A 3% w/v agarose solution was applied to young cotyledons, peeled off after hardening and analyzed under the light microscope.

### Microscopical Methods

Stereo microscopy was carried out with the Leica MZ 16F stereo microscope and the LAS AF software. Light and fluorescence microscopy was carried out with the Leica DMRB microscope equipped with a Sony Alpha 6000 camera or with the Leica DMRE microscope equipped with the Leica DFC 7000 T camera using the LAS X software. Confocal laser scanning microscopy was carried out with the Leica DM5500 and DM6000 CS Microscopes and documented with the TCS-SPE and TCS-SP8 imaging systems, respectively (Leica Microsystems, Heidelberg, Germany).

### Transcript Analysis

Total RNA was isolated (TRI Reagent, Ambion by Life Technologies) and treated with DNaseI (Thermo Fisher Scientific). Integrity of RNA was confirmed on a bleach gel (Aranda et al., 2012) and the concentration was quantified spectrophotometrically. One microgram of total RNA was reverse-transcribed (SuperScriptIII, Invitrogen).

qPCRs were carried out in a QuantStudio 5 System (ABI/Life Technologies) equipped with a 96 well block. The qPCRs were performed using plates (96 well, 0.2 ml) and cover foil (Opti-Seal Optical Disposable Adhesive, BIOplastics) and SYBR Green reagent (Thermo Fisher Scientific). All results represent the average of three biological and three technical replicates. Analysis was carried out with the QuantStudio TM Design and Analysis Software version 1.4.1 and Excel 2007. Outliers were removed using a two-sided Grubbs test at a significance level of 0.05. Efficiency of primers was calculated in cDNA dilution series of 1:10, 1:20, 1:40, 1:80, 1:160, and 1:320. Higher dilutions were not possible due to the concentration of RNA/cDNA. Primers for reference genes were described before (Wang et al., 2009; Stephan et al., 2019). Primers for genes of interest were accepted with an efficiency of 80–120% and a correlation between −1 and −0.99 (Supplementary Table S1). Normalization against one reference gene was carried out using the ΔΔCt method. Normalization against two reference genes was carried out using normalization factors, according to the geNorm manual (Vandesompele et al., 2002). Standard deviations between biological replicates were calculated over the means of the single replicates, rather than the raw data.

### Sequence Analysis, Plant Measurements, Statistical Analysis

Sequences were taken from the Genomic resources for *Arabis alpina* website^1^. *In silico* sequence analysis was carried out with CLC DNA Workbench version 5.6.1. Conserved domains were determined using CD-Search (Marchler-Bauer and Bryant, 2004) and/or PROSITE (Hulo et al., 2007). Phylogenetic trees were created using the Neighbor Joining method with the Unipro UGENE software (version 1.16.0) and subsequently modified for clarity using Adobe Illustrator CS4 version 14.0.0.

All plant measurements were carried out using ImageJ (Fabrice Cordelieres, Institut Curie, Orsay, France). Data was subsequently analyzed with Microsoft Excel 2007. Cell complexity was calculated using the formula $c\,o\,m\,p\,l\,e\,x\,i\,t\,y=\frac{p\,e\,r\,i\,m\,e\,t\,e\,r^{2}}{4\times \pi \times a\,r\,e\,a}$.

Statistical analysis was carried out with OriginPro 8.5 0G SR0, Microsoft Excel 2007 or R Studio. Significance levels were tested as indicated in the results section.

### Plasmids

The coding sequences of AaSPI-PBW (Aa_G228370), AaDCP1 (Aa_G368370), AaSKD1 (Aa_G151760), AaLIP5 (Aa_G86640), AaVPS60.1 (Aa_G137100), AaVPS60.2 (Aa_G304080) were amplified from cDNA of *pep1* (Supplementary Table S1). Gateway donor or destination vectors containing AtSPI-PBW (AT1G03060), AtDCP1 (AT1G08370), AtDCP2 (AT5G13570), AtDCP5 (AT1G26110), AtSKD1 (AT2G27600), AtLIP5 (AT4G26750), AtVPS60.1 (AT3G10640), AtVPS60.2 (AT5G04850), AtTTG1 (AT5G24520), AtGL3 (AT5G41315), and AtMYC1 (AT4G00480) have been described before (Zimmermann et al., 2004; Shahriari et al., 2010; Zhao et al., 2012; Steffens et al., 2014, 2015; Tian et al., 2015). Gateway vectors pENSG:YFP/CFP, pEXSG:YFP/CFP (Feys et al., 2005) and pAMARENA (M. Jakoby, NCBI:txid905036) were used for localization, pSYN/pSYC (Jakoby et al., 2006) and pCL112/113 (provided by J. F. Uhrig) were used for BiFC, pACT/pAS (Clonetech) were used for yeast two-hybrid analyses.

### Protein–Protein Interaction Assays and Co-localization

Yeast two-hybrid assays were carried out as described previously (Gietz et al., 1995). The selection on synthetic dropout interaction media lacking leucine (-L), tryptophan (-W), and histidine (-H), was performed with 3-aminotriazole (3AT) concentrations up to 30 mM. Bimolecular Fluorescence Complementation (BiFC) analysis was done in Arabidopsis leaves by biolistic transformation as described before (Mathur et al., 2003). Comparability of BiFC samples was guaranteed by fixed laser intensities, gain and line accumulation. The raw data was enhanced for clarity using Adobe Photoshop CS4 version 14.0.0 and ImageJ (Fabrice Cordelieres, Institut Curie, Orsay, France). Co-localization of AaSPI-PBW with ESCRT and P-body components was carried out in leaves of 3 week old Arabis seedlings by biolistic transformation (Mathur et al., 2003).

## Results

### *Arabis alpina spi* Mutants and *SPI* Expression

**FIGURE 1:**
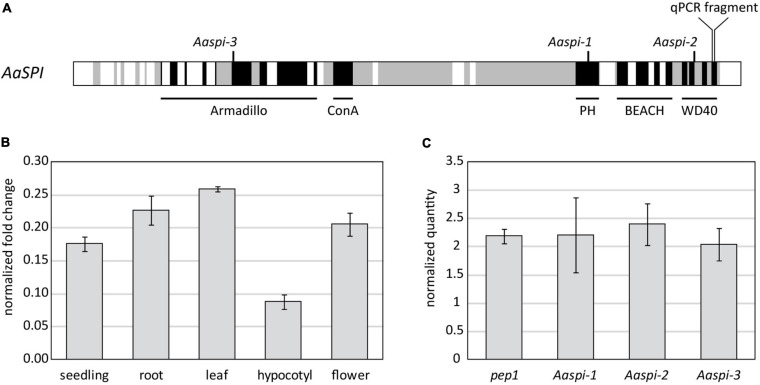
Molecular characterization of the *SPIRRIG* gene in *Arabis alpina*. **(A)** Schematic presentation of *AaSPIRRIG*. Exons are shown in gray, functional domains are depicted in black. The positions of early stop codons within the *spirrig* alleles are indicated above. **(B)** The expression levels of *AaSPI* were determined in five tissues by qPCR. Normalization was carried out using *AaRAN3* (Wang et al., 2009). **(C)** The expression levels of *AaSPI* were determined in the above ground parts of seedlings of *pep1*, *Aaspi-1*, *Aaspi-2*, and *Aaspi-3* by qPCR. Normalization was carried out using *AaTUA5* and *AaCAC* (Stephan et al., 2019).

Phylogenetic analysis has shown that BEACH domain proteins can be clustered into four groups (A–D), each containing plant as well as animal genes (Saedler et al., 2009). The Arabidopsis *SPI* gene belongs to group A, which includes animal genes that were described to be involved in membrane trafficking (De Lozanne, 2003). In our phylogenetic analysis, we found the *SPI* gene well conserved in all plant species analyzed (Supplementary Figure S1). In *Arabis alpina*, the orthologous *SPI* gene was identified by sequence homology and the synteny of homologous genes on the chromosome (Chopra et al., 2019). Three *spi* mutants were identified in an EMS mutagenesis screen for trichome phenotypes (Chopra et al., 2019) in the *perpetual flowering 1* (*pep1*) mutant, in the pajares (paj) ecotype background (Wang et al., 2009). This line harbors a mutation in the floral repressor *PEP1*, which causes the plants to flower without vernalization (Albani et al., 2012). Therefore, working in the *pep1* background greatly facilitates the genetic analysis of processes not related to flowering. All three alleles display early STOP codons (Figure 1A). To verify that the three mutant lines are indeed three *spi* alleles we performed complementation tests. F1 plants from pairwise crosses between the three mutants showed a severe trichome phenotype indicating the mutations in the *SPI* gene are causative for the observed trichome phenotype (Supplementary Figure S2).

The expression profile of *AaSPI* was determined in whole seedlings, roots, mature leaves, hypocotyls and flowers (Figure 1B). The transcript is expressed throughout the whole plant, similar as observed in *Arabidopsis thaliana* (Saedler et al., 2009). The expression is lower in the hypocotyl, compared to other tissues of the plant. The expression levels in the three mutant alleles are similar to that in *pep1*, which is used as the wild-type reference for *AaSPI* (Figure 1C). This shows that the aberrant transcripts are not recognized and degraded by non-sense mediated decay and that the truncation of the protein is the cause for the mutant phenotype.

### *Aaspi* Mutants Display Trichome-, Root-, and Epidermal Pavement Cell Phenotypes

**FIGURE 2:**
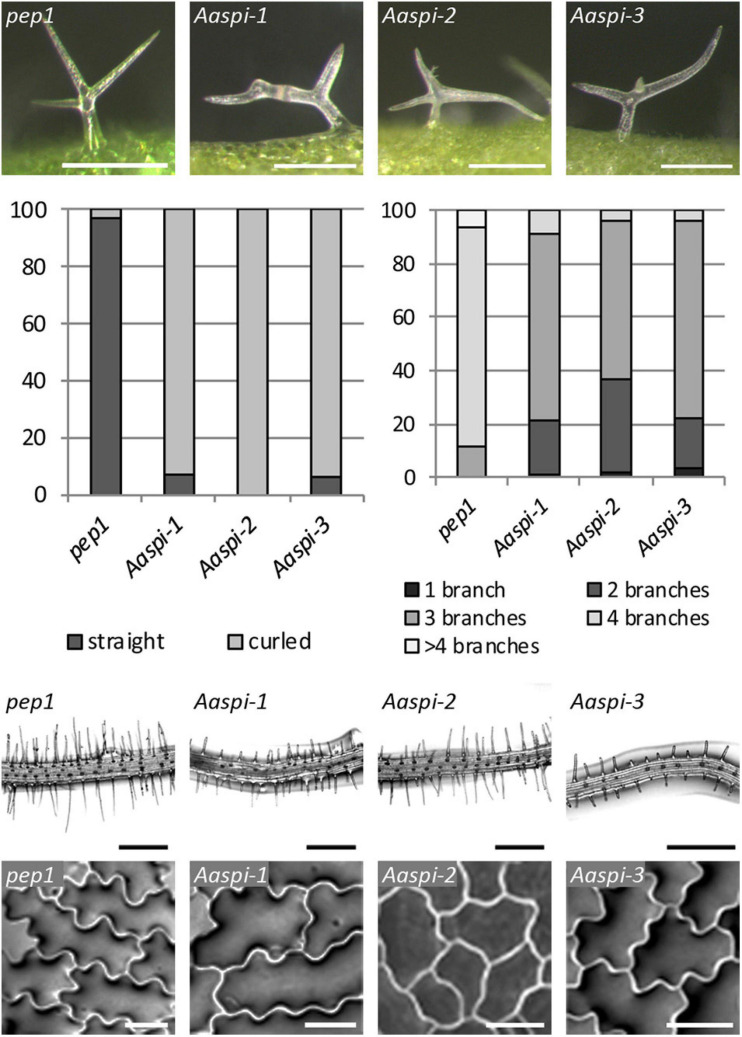
The *Aaspi distorted* phenotype of trichomes, roots and epidermal pavement cells. **First row:** Microscopic pictures of distorted trichomes. **Second row,** left: Ratio of straight and curled trichomes in *pep1* and *Aaspi* mutants. Second row, right: Number of trichome branches in *pep1* and *Aaspi* mutants. Graph shows the relative abundance in% in more than 100 trichomes per line. **Third row:** Microscopic images of root hair phenotypes to illustrate the shorter root hairs in the *Aaspi* mutants. Scale bare: 300 μm. **Bottom row:** Microscopic images of epidermal pavement cells to illustrate the lower complexity of epidermal pavement cells on cotyledons. Scale bar: 50 μm.

The three *Aaspi* alleles had been identified by their weak *distorted* trichome phenotype (Chopra et al., 2019; Figure 2). In Arabidopsis, it was shown that *spi* mutant trichomes exhibit a reduced stalk length and width and a reduced average branch length (Saedler et al., 2009). In addition, we found that Arabidopsis *Atspi* mutants have a decreased number of branches (Supplementary Table S2). To compare the phenotypic spectrum of the *Aaspi* mutants with that in *Atspi* mutants, we studied the trichome phenotype in more detail. We found that almost all branches were curled or *distorted* in *Aaspi* mutants (Figure 2). Stalk length was significantly reduced in all three *Aaspi* alleles. Also, trichome branching was clearly reduced in the three *Aaspi* mutants (Figure 2). Stalk width and branch length varied in the three alleles such that some of the *Aaspi* alleles showed a difference to *pep1* and others not (Table 1). We therefore considered these two aspects of the *distorted* phenotype as not affected by the *Aaspi* mutations.

**TABLE 1:** Cell complexity, root hair length and trichome measurements in *pep1* and *Aaspi* mutants.

	***pep1***	***Aaspi-1***	***Aaspi-2***	***Aaspi-3***
Complexity	2.75 ± 0.56	2.36 ± 0.45***	2.01 ± 0.57***	2.16 ± 0.42***
Root hair length (μm)	188 ± 91	95 ± 32***	108 ± 41***	89 ± 31***
Trichome stalk length (μm)	143 ± 59	10 ± 72***	4 ± 41***	88 ± 65***
Trichome stalk width (μm)	26 ± 7	26 ± 8	22 ± 5***	25 ± 7
Trichome branch length (μm)	159 ± 59	163 ± 75	122 ± 52***	153 ± 69

*Mean complexity of 50 cells per line (10 cells on 5 leaves) was calculated with the formula Complexity = perimeter/(4⋅Π⋅area) (Dewitte and Murray, 2003). Significance was tested with a one way ANOVA at a significance level of 0.001 (^∗∗∗^), 0.02 (^∗∗^), and 0.05 (^∗^). The individual length of 150 seven day old root hairs per line was measured 1 mm below the intersection of hypocotyl and root for 15 root hairs per sample. Significance of the data was calculated with a Wilcoxon signed-rank test at a significance level of 0.001 (^∗∗∗^), 0.02 (^∗∗^), and 0.05 (^∗^). The stalk length is defined as the distance between leaf surface and the first branch, the stalk width was measured at the middle point of the stalk. The table shows the mean and standard deviation of 100 measurements per line and trichome parameter on mature leaves. Significance of the data was calculated with a Wilcoxon signed-rank test at a significance level of 0.001 (^∗∗∗^), 0.02 (^∗∗^), and 0.05 (^∗^).*

Arabidopsis *spi* mutants are characterized by morphological changes in other cell types as well. In *Atspi* mutants root hairs are shorter as compared to wild-type (Saedler et al., 2009). We analyzed *Aaspi* mutant root hairs about 1 mm below the border between hypocotyl and the root of 7 days old seedlings. We found that *Aaspi* mutants also have significantly shorter root hairs than *pep1* (Figure 2 and Table 1).

In Arabidopsis, *Atspi* epidermal pavement cells show less lobing and, as a result, less complexity than wild-type cells (Saedler et al., 2009). To assess whether this phenotype is also found in *Aaspi* mutants, we inspected the pavement cells on cotyledons. We show that *Aaspi* mutant epidermal cells are less lobed than *pep1* cells (Figure 2). To quantify the difference in the lobing of cells we determined the mean complexity as done before (Dewitte and Murray, 2003) and found a significant reduction in all three *Aaspi* alleles (Table 1).

### Hypocotyl Growth Is Not Reduced in *Aaspi* Mutants

In Arabidopsis, young *spi* seedlings show an almost twofold reduction of the hypocotyl length (Saedler et al., 2009). Our analysis of *Aaspi* seedlings revealed no decrease of hypocotyl length as compared to *pep1* (Table 2). If at all, hypocotyls of *Aaspi* seedlings were longer as observed in *Aaspi-2* and *Aaspi-3*. We also never observed individual hypocotyl cells tearing out of the epidermal cell surface when grown in the dark. These observations correlate with our finding that *AaSPI* expression is relatively lower in the hypocotyl as compared to Arabidopsis, suggesting that AaSPI is less important for hypocotyl growth.

**TABLE 2:** The hypocotyl length of *pep1* and *Aaspi* mutants.

	***pep1***	***Aaspi-1***	***Aaspi-2***	***Aaspi-3***
Hypocotyl length (cm)	2.27 ± 0.46	2.43 ± 0.55	2.69 ± 0.47***	2.56 ± 0.47**

*The hypocotyl length was measured in 50 seven day old seedlings per line. Significance of the data was calculated with a Wilcoxon signed-rank test at a significance level of 0.001 (^∗∗∗^), 0.02 (^∗∗^), and 0.05 (^∗^).*

### *AaSPI* Is Not Important for Vacuolar Integrity in *Arabis* Root Hairs

**FIGURE 3:**
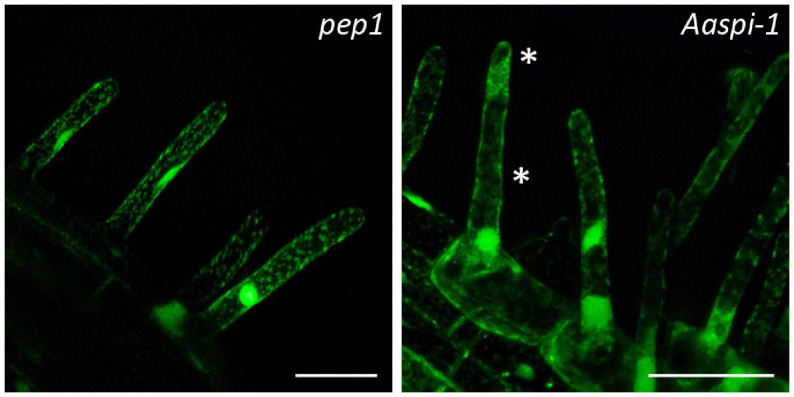
Fragmented root hair vacuoles in *pep1* and *Aaspi-1* mutants. Left: Representative picture of an intact vacuole in *pep1*. Right: Representative picture of a fragmented vacuole in *Aaspi-1*. Asterisks indicate fragmented vacuoles. Root hairs were stained with FDA and integrity of vacuoles was analyzed in > 100 root hairs per line. Scale bars: 50 μm.

**FIGURE 4:**
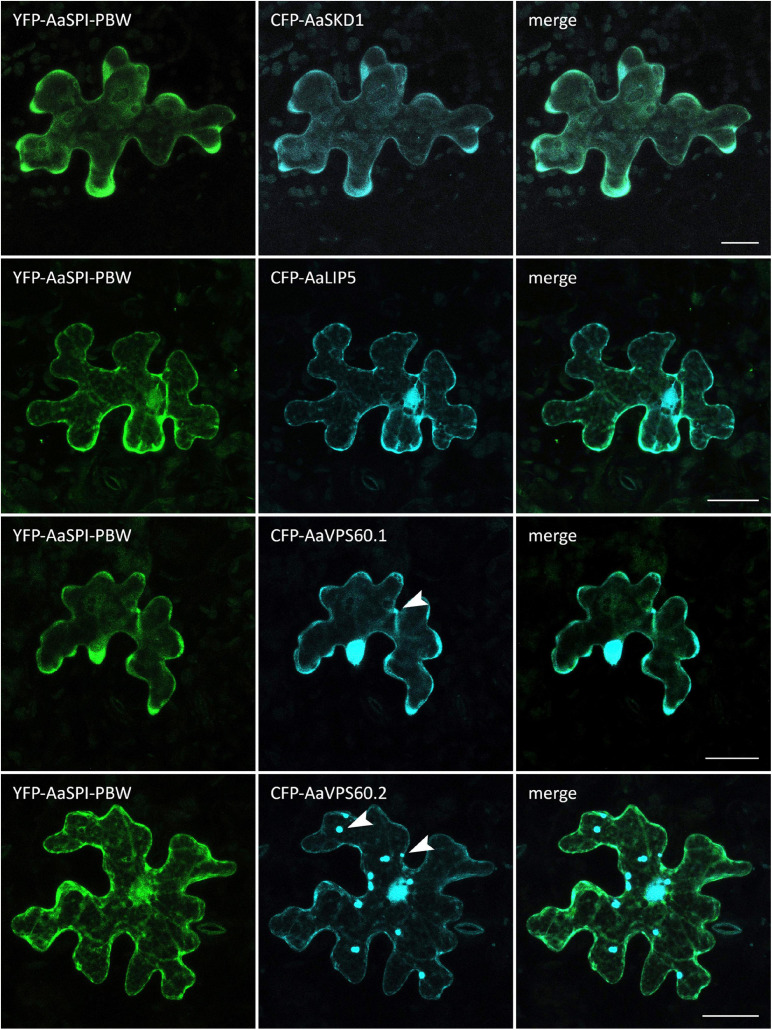
Intracellular localization of CFP-AaESCRT components and YFP-AaSPI-PBW. Transiently expressed CFP tagged AaESCRT proteins and YFP-AaSPI-PBW in *Arabis alpina* epidermal pavement cells. Arrow heads indicate dot-like structures. Scales bars: 25 μm.

**FIGURE 5:**
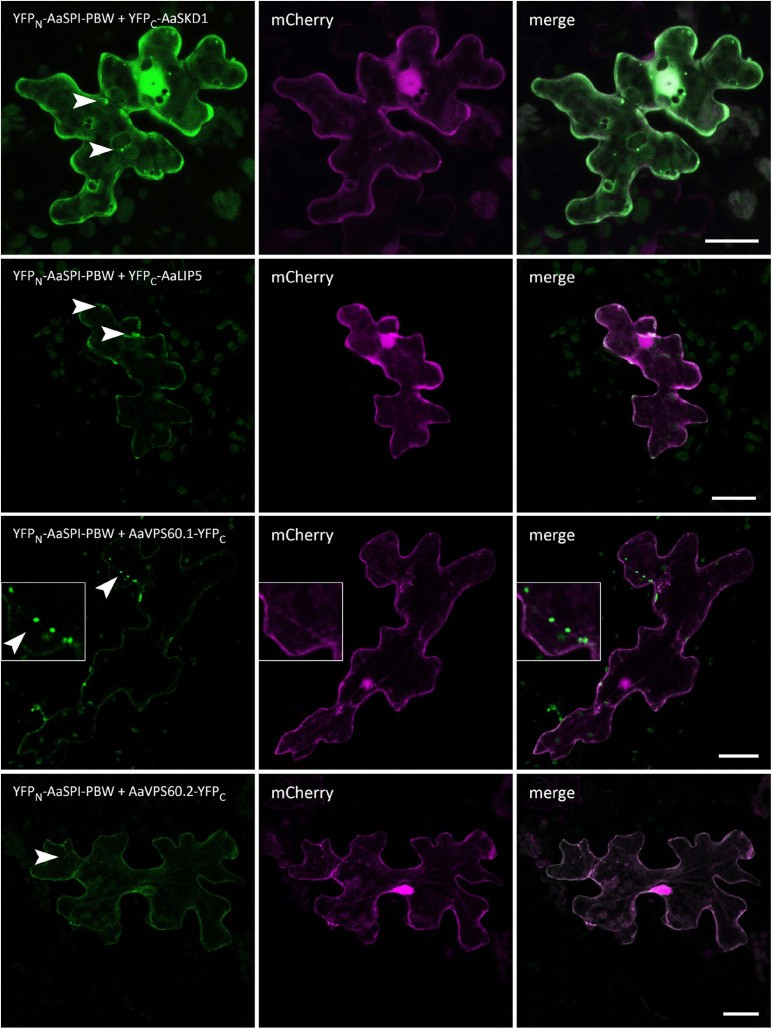
BiFC interactions of AaSPI-PBW with ESCRT components AaSKD1, AaLIP5, AaVPS60.1, and AaVPS60.2. Left—BiFC, middle—transformation control, right—overlay of BiFC and transformation control. Arrow heads indicate the sites of interaction. Scales bars: 25 μm.

**FIGURE 6:**
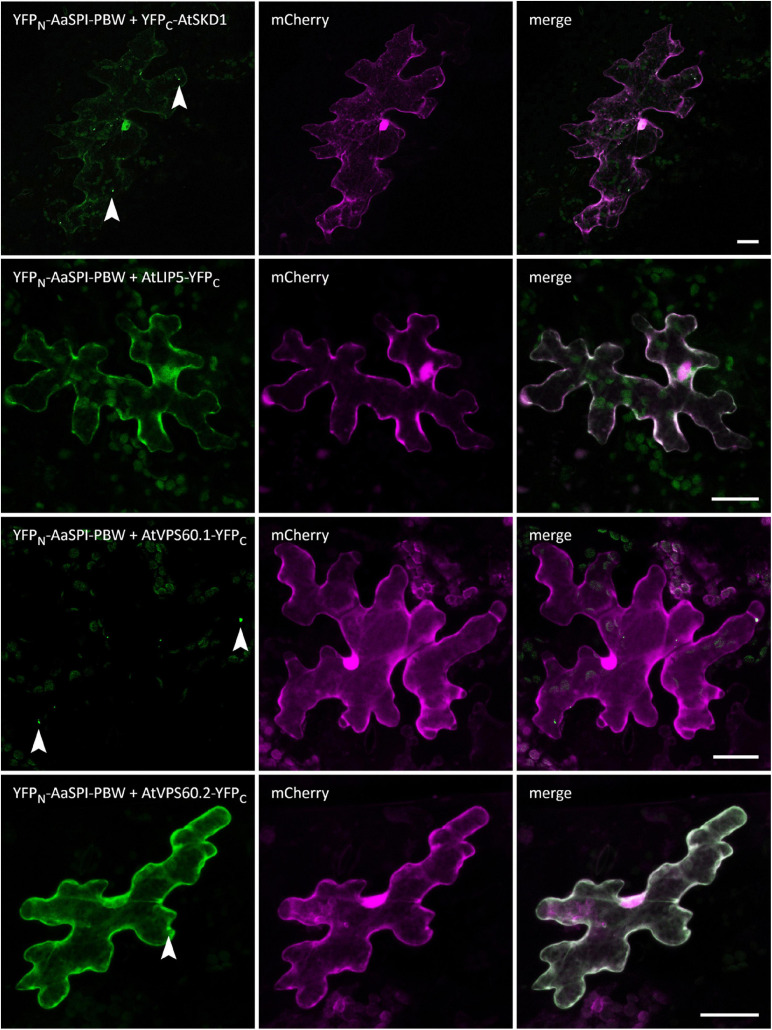
BiFC interactions of AaSPI-PBW with ESCRT components AtSKD1, AtLIP5, AtVPS60.1, and AtVPS60.2. Left—BiFC, middle—transformation control, right—overlay of BiFC and transformation control. Arrow heads indicate sites of interaction. Scale bars: 25 μm.

In Arabidopsis root hairs vacuoles were found to be fragmented, giving the initial hint toward a function of AtSPI in membrane trafficking/organization similar as reported for BEACH domain proteins in other species (Saedler et al., 2009). Toward this end we analyzed Fluorescein Diacetate (FDA) stained root hairs of *pep1* and *Aaspi* mutants. Our analysis revealed a low frequency of root hairs with fragmented vacuoles in the range of 5% in both *pep1* and all *Aaspi* mutants. This indicates that *Aaspi* mutants are not affected in vacuolar integrity of root hairs (Figure 3 and Table 3). This finding raised the question, whether the AaSPI protein interacts with ESCRT proteins, similar as reported for AtSPI (Steffens et al., 2017). In a first step, we studied the intercellular localization by transiently transforming *Arabis alpina* epidermal cells with YFP tagged proteins. In these experiments, YFP-AaSPI localized to the cytoplasm (Figure 4). Also, the Arabis ESCRT components CFP-AaSKD1 and CFP-AaLIP5 were found in the cytoplasm. CFP-AaVPS60.1 and CFP-AaVPS60.2 were found in the cytoplasm and in dot-like structures (Figure 4). In a second step, we studied the interaction of the AaSPI-PBW fragment with the four ESCRT proteins from *A. alpina*. In addition, we tested the interaction of AaSPI-PBW with ESCRT proteins from *A. thaliana* to assess the interspecies interactions (Table 4). In these experiments we found interactions between AaSPI-PBW and all Arabidopsis and Arabis ESCRT proteins, similar as reported for Arabidopsis SPI-PBW. To study the interaction behavior in *Arabis alpina* cells, we performed Bimolecular Fluorescence Complementation (BiFC) experiments. BiFC interaction was found between AaSPI and all ESCRT proteins from Arabis and Arabidopsis (Figures 5, 6). Positive and negative BiFC controls are shown in Supplementary Figures S5–S7. It would be desirable to further clarify the interaction behavior of AaSPI, however, also pull-down assays failed in our hands. Assays with bacterially expressed proteins did not work since we could not recover sufficient amounts of soluble proteins.

**TABLE 3:** Vacuole integrity in *pep1* and *Aaspi* mutants.

	***pep1***	***spi-1***	***spi-2***	***spi-3***
Intact vacuoles (%)	93.58	94.17	93.16	93.79
fragmented vacuoles (%)	6.42	5.83	6.84	6.21

*Root hairs were stained with FDA and integrity of vacuoles was analyzed in > 100 root hairs per line.*

**TABLE 4:** Yeast two-hybrid interaction between AaSPI-PBW and ESCRT components.

	**GAL4BD**							
	
	***Arabis alpina***				***Arabidopsis thaliana***			
		
**GAL4-AD**	**LIP5**	**SKD1**	**VPS60.1**	**VPS60.2**	**LIP5**	**SKD1**	**VPS60.1**	**VPS60.2**
AaSPI-PBW	++	++	+++	+++	+++	+	+++	+++

*Interaction between AaSPI-PBW, N-terminally fused to the GAL4-AD, and ESCRT components from Arabis alpina and Arabidopsis thaliana, N-terminally fused to the GAL4-BD, was tested on selective dropout medium lacking Leu, Trp, and His, supplemented with increasing concentrations of 3-aminotriazole (3AT). GFP, N-terminally fused to the GAL4-AD, was included as a negative control. The number of plus letters indicates the strength of the interaction [growth on selective media (+), with up to 15 mM 3AT above control (++), with up to 25 mM 3AT above control (+++)].*

### Interaction Between AaSPI and the P-Body Component DCP1

**FIGURE 7:**
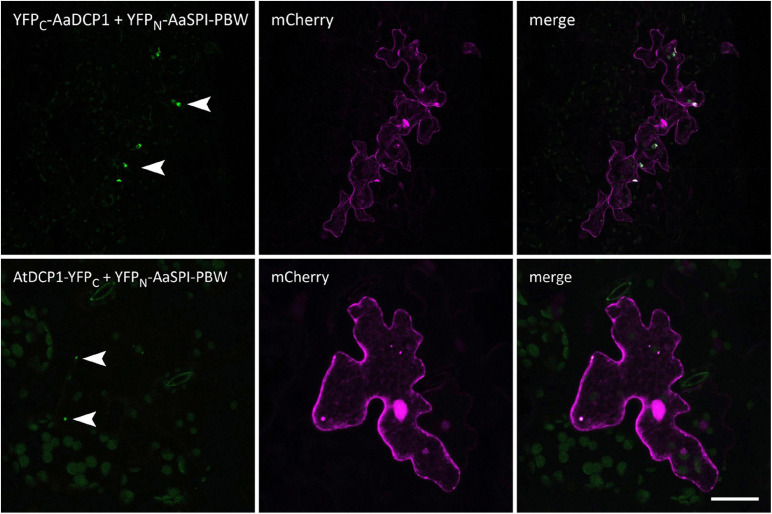
BiFC interactions of AaSPI-PBW with the P-body component DCP1 from *A. thaliana* and *A. alpina*. Left—BiFC, middle—transformation control, right—overlay of BiFC and transformation control. Arrow heads indicate sites of interaction. Scale bars: 25 μm.

The PBW domain of AtSPI had been shown to interact with DCP1 homologs from mammals and yeast (Steffens et al., 2015). To determine, whether this behavior is conserved in *A. alpina*, we studied the interaction of AaSPI with DCP1 in yeast two-hybrid and BiFC assays. We found interactions with the AaDCP1 and AtDCP1 proteins (Table 5 and Figure 7).

**TABLE 5:** Yeast two-hybrid interaction between AaSPI-PBW and P-body components.

	**GAL4BD**			
	
	***Arabis alpina***		***Arabidopsis thaliana***	
		
**GAL4-AD**	**DCP1**	**DCP2**	**DCP1**	**DCP2**
AaSPI-PBW	+	−	+	−

*Interaction between AaSPI-PBW, N-terminally fused to the GAL4-AD, and P-body components from Arabis alpina and Arabidopsis thaliana, N-terminally fused to the GAL4-BD, was tested on selective dropout medium lacking Leu, Trp, and His, supplemented with increasing concentrations of 3-aminotriazole (3AT). GFP, N-terminally fused to the GAL4-AD, was included as a negative control. The number of plus letters indicates the strength of the interaction [no growth on selective media (−); growth on selective media with < 15 mM 3AT above control (+)].*

**FIGURE 8:**
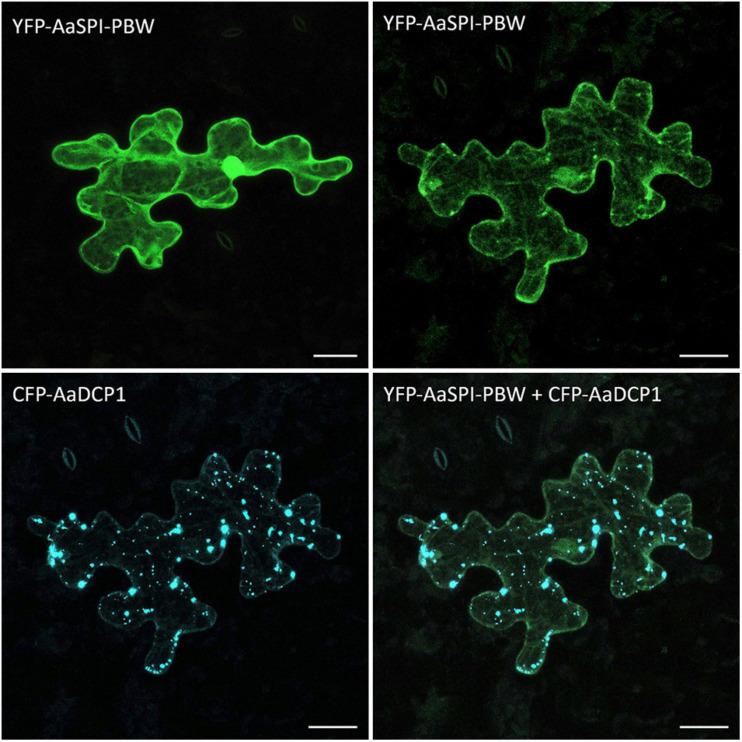
Localization of AaSPI-PBW in *pep1* epidermal pavement cells. Transient expression of YFP-AaSPI-PBW (first row), cytosolic (left) and cytosolic with dots (right); and CFP-AaDCP1 (second row), in dots (left) and in dots overlapping the expression of YFP-AaSPI-PBW. Scale bars: 25 μm.

In Arabidopsis, SPI-PBW co-localization to DCP1 is triggered by salt stress, suggesting that this stress treatment causes a re-localization of the AtSPI-PBW protein (Steffens et al., 2015). In a first step we studied the co-localization of AaSPI-PBW and AaDCP1 under normal conditions. AaSPI-PBW alone was evenly distributed in the cytoplasm (Figure 8). When co-expressed with AaDCP1, AaSPI-PBW appeared in the cytoplasm and additionally in AaDCP1-marked P-bodies in the majority of cells (Figure 8). Thus, AaDCP1-PBW is recruited to P-bodies under normal conditions in these experiments. Upon salt stress (up to 300 mM of NaCl) the majority of leaf epidermal cells did not show a response to the treatment. However, we observed an increased number of small P-bodies and additional recruitment of AaSPI-PBW to AaDCP1-marked P-bodies in very few cells after 40 min of salt treatment (Supplementary Figure S3).

### The Salt Stress Phenotype of Arabis *spi* Alleles

We analyzed the salt response of *Aaspi* mutants, as SPI was shown to be essential for the salt stress tolerance of Arabidopsis (Steffens et al., 2015). We tested effects of salt on germination, primary root growth and seedling growth, representing different tissues and stages of development.

**FIGURE 9:**
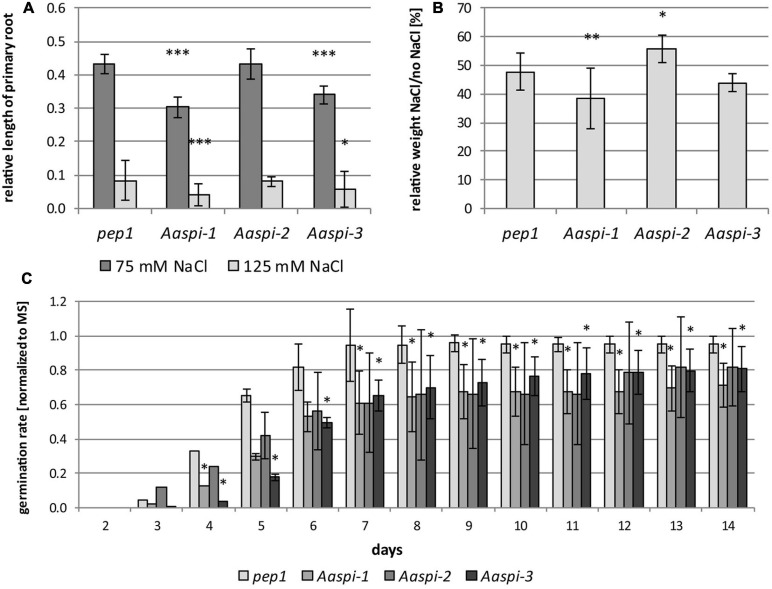
Salt treatments in *pep1* and *Aaspi* alleles. **(A)** Primary root growth of *pep1* and *Aaspi* on different concentrations of NaCl after 4 days. Primary root growth under salt stress conditions was tested in three independent experiments. The assays were carried out on plates containing 0 mM NaCl, 75 mM NaCl and 125 mM NaCl, respectively. Roots were measured after 4 days of growth. Significance of the data was tested using an ANOVA with *p* < 0.001 (***), *p* < 0.01 (**), and *p* < 0.05 (*). Total N per line per condition 93–128. **(B)** Seedling growth of *Arabis alpina* lines on soil under salt stress. Plants were watered every 3–4 days with water for the first 20 days, subsequently plants were watered with either water or water with 125 mM NaCl (*N* = 27–31 per line and condition). The weight of the seedlings was determined after 30 days. Significance of the data was tested using ANOVA with *p* < 0.001 (***), *p* < 0.01 (**), and *p* < 0.05 (*). **(C)** Germination of *pep1* and *spi* mutants under salt stress. Germination of *pep1* and the three *Aaspi* mutants was analyzed on MS plates and MS plates supplemented with 125 mM NaCl in three independent sets (*N* > 115 per line and condition). The relative number of seeds germinated on salt media was normalized against the values from media containing no salt and combined between the sets. Error bars depict standard deviations. Significance was tested using Mann-Whitney-U at a level of *p < 0.10.

Germination and primary root growth were significantly inhibited in *Aaspi-1* and *Aaspi-3* (Figures 9A,C). Strikingly, *Aaspi-2* did not show an altered salt response compared to *pep1*. At a later stage of development and under transpiring conditions, salt hypersensitivity is only visible for *Aaspi-1* (Figure 9B). The *Aaspi-3* allele is indistinguishable from *pep1* at this stage of development, while *Aaspi-2* is hyposensitive to salt.

**FIGURE 10:**
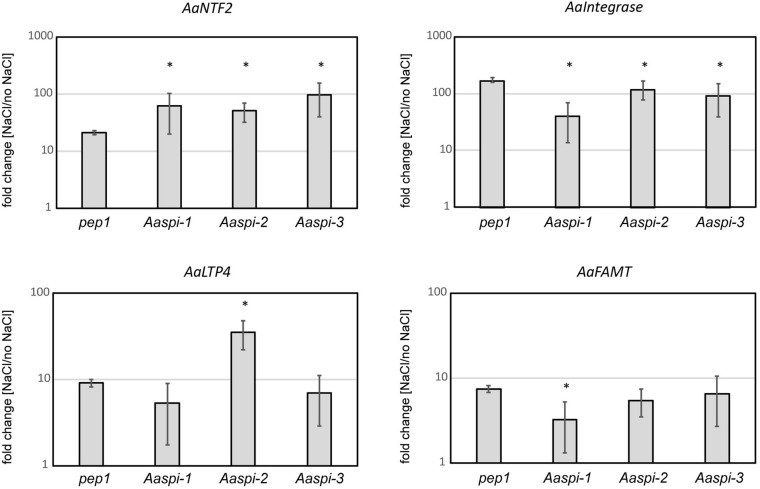
Relative transcript levels in *pep1* and *Aaspi* mutants in response to salt stress. Normalization was carried out against *AaCAC* and *AaTUA5*. Error bars depict standard deviation. Significance was determined using a Mann-Whitney-U test at *p < 0.10.

On the transcript level, we identified four genes which are differentially regulated under salt stress in *Aaspi* mutants (Figure 10). These genes were chosen following an RNAseq analysis in *A. thaliana* (Steffens et al., 2015). Two of which showed a similar salt response of all *Aaspi* alleles (Aa_G78660/AT3G07250 and Aa_NA/AT1G71520), while the other two reflected the phenotypic differences between the alleles (Aa_G499040/AT5G59310 and Aa_NA/AT3G44860). AT3G07250 is the nuclear transport factor family protein AtNTF2. This protein has a function in RNA binding and is likely to function as a ribonucleoprotein complex (Lamesch et al., 2012). *AaNTF2* mRNA levels are strongly increased under salt stress, with a significantly higher upregulation in all three *Aaspi* mutants, compared to *pep1* (Figure 10). AT1G71520 is an integrase-type DNA-binding superfamily protein with DNA-binding transcription factor activity, which is involved in ethylene-activated signaling, regulation of transcription and response to chitin (Lamesch et al., 2012). Under salt conditions, all *Aaspi* mutants showed a decreased upregulation compared to *pep1* (Figure 10). AT5G59310 encodes the lipid transfer protein 4 (AtLTP4). Proteins of this family can bind fatty acids and acylCoA esters. They can transfer phospholipids and function in response to abscisic acid, salt stress and drought (Lamesch et al., 2012). For this gene, *Aaspi-2* showed a significantly higher upregulation in salt conditions compared to *pep1* and the other two *Aaspi* alleles (Figure 10), possibly explaining the hyposensitivity to salt in later developmental stages. AT3G44860 encodes FARNESOIC ACID CARBOXYL-O-METHYLTRANSFERASE (AtFAMT). The role of farnesoic acid in plants has not been revealed so far, however, the transcript level of FAMT is increased upon various stresses, indicating that it might have general functions in stress responses (Yang et al., 2006). The qPCR results showed that the increase of transcript was significantly less in *Aaspi-1* compared to *pep1* and the other two *spi* alleles (Figure 10), which could explain the strong hypersensitivity of this allele.

## Discussion

Our phenotypic analysis of Arabis *spi* mutants revealed that they share most of the morphological phenotypes with Arabidopsis *spi* mutants. We also found a similar localization and protein-protein interaction behavior of the AtSPI and AaSPI proteins with ESCRT proteins. Taking these observations together, it is conceivable that the regulation of cell morphogenesis and a molecular function in the endosomal pathways is conserved in the two species. In support of this, also *spi* mutants of the moss *Marchantia polymorpha* exhibit short rhizoids reminiscent to the root hair phenotype of *Aaspi* and *Atspi* (Honkanen et al., 2016).

Interestingly, we also found that Arabis *spi* alleles show a different response to salt stress than *pep1*, suggesting that the dual function of SPI in morphogenetic processes and physiological processes is conserved between the two species. The molecular function of AaSPI in salt stress responses and mRNA regulation remains unclear. We found a physical interaction of AaSPI with AtDCP1 and AaDCP1. However, we cannot decide, whether the salt-dependent localization of cytoplasmic SPI protein to P-bodies as observed in Arabidopsis (Steffens et al., 2015) takes place in Arabis as co-expression of DCP1 and SPI is sufficient to trigger a recruitment of SPI to P-bodies. We also found transcriptional changes of several salt-stress response genes when comparing *Aaspi* alleles and *pep1*. Whether RNA stability is changed in *Aaspi* mutants was not tested in this study.

It is also not clear, whether AaSPI plays a role in salt stress responses. A salt stress phenotype is seen in *Aaspi-1* and *Aaspi-3* but not in *Aaspi-2.* One way to interpret this is to conclude that AaSPI is not involved in salt stress response because not all alleles show the phenotype. In this scenario, background mutations in *Aaspi-1* and *Aaspi-3* would cause the salt stress phenotype. Alternatively, it is possible that the different behavior of the three alleles is due to the type of mutations that they carry. While in *Aaspi-1* and *Aaspi-3* alleles premature stop codons lead to a loss of the PH-BEACH domain, the stop codon in *Aaspi-2* leads to a truncation of only two of the four WD40 repeats in the C-terminus. Thus, it is possible that the protein region between the stop codons is relevant for the salt stress response. In this scenario it would be possible to assign protein domains that would be responsible for salt stress responses but not for cell morphogenesis.

## Data Availability Statement

The original contributions presented in the study are included in the article/Supplementary Material, further inquiries can be directed to the corresponding author/s.

## Author Contributions

LS, MJ, and MH designed this study and wrote the manuscript. LS performed the research and analyzed the data. MH directed the project. All authors contributed to the article and approved the submitted version.

## Conflict of Interest

The authors declare that the research was conducted in the absence of any commercial or financial relationships that could be construed as a potential conflict of interest.

## Footnotes

1 www.arabis-alpina.org
