# Supplementary material for: Evolutionary Comparison of the Developmental/Physiological Phenotype and the Molecular Behavior of SPIRRIG Between Arabidopsis thaliana and Arabis alpina
Source: Front Plant Sci. 2021 Jan 7;11:596065. doi: 10.3389/fpls.2020.596065 (PMC7874212; doi:10.3389/fpls.2020.596065)
Supplement: Supplementary file 1 [file presentation_1.pptx]

## Slide 1
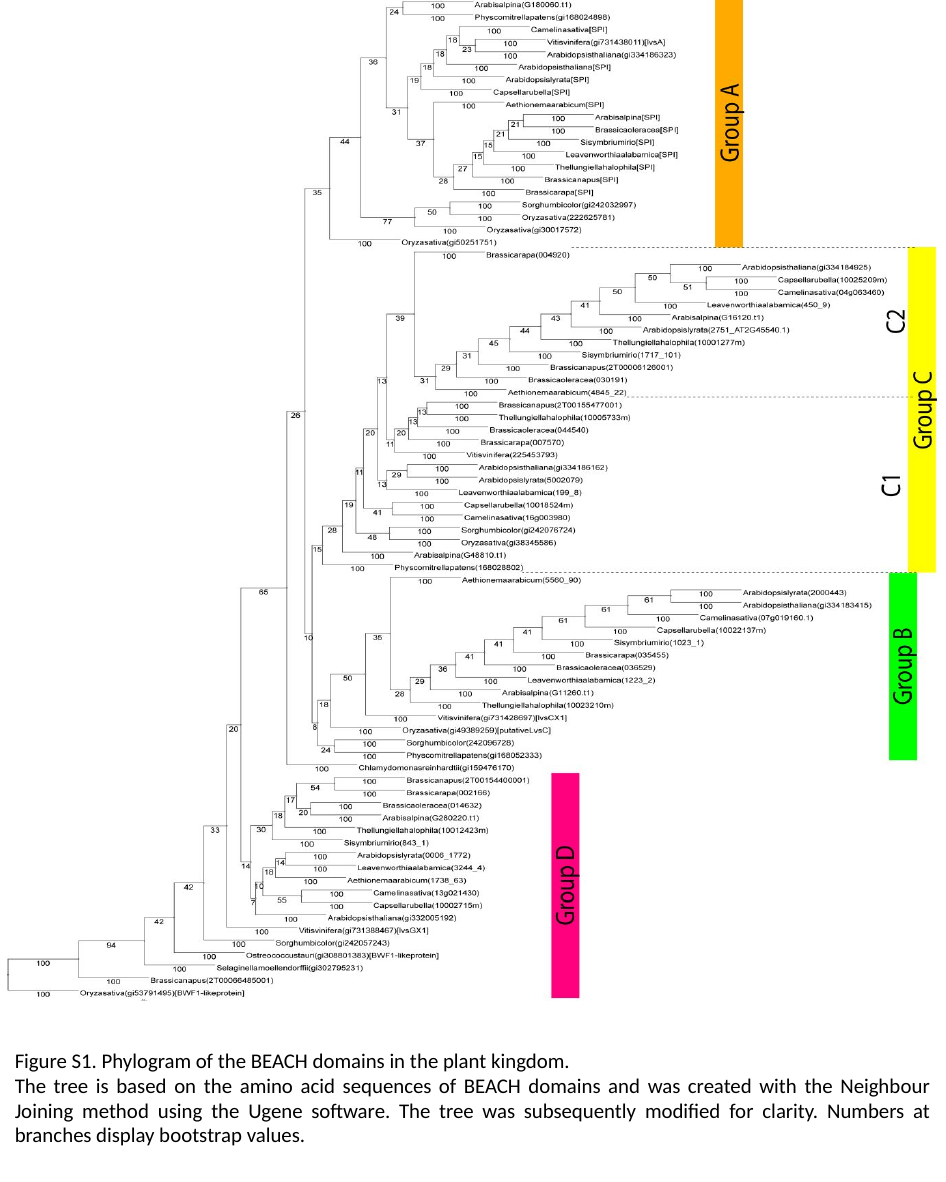

Figure S1. Phylogram of the BEACH domains in the plant kingdom.
The tree is based on the amino acid sequences of BEACH domains and was created with the Neighbour Joining method using the Ugene software. The tree was subsequently modified for clarity. Numbers at branches display bootstrap values.

## Slide 2
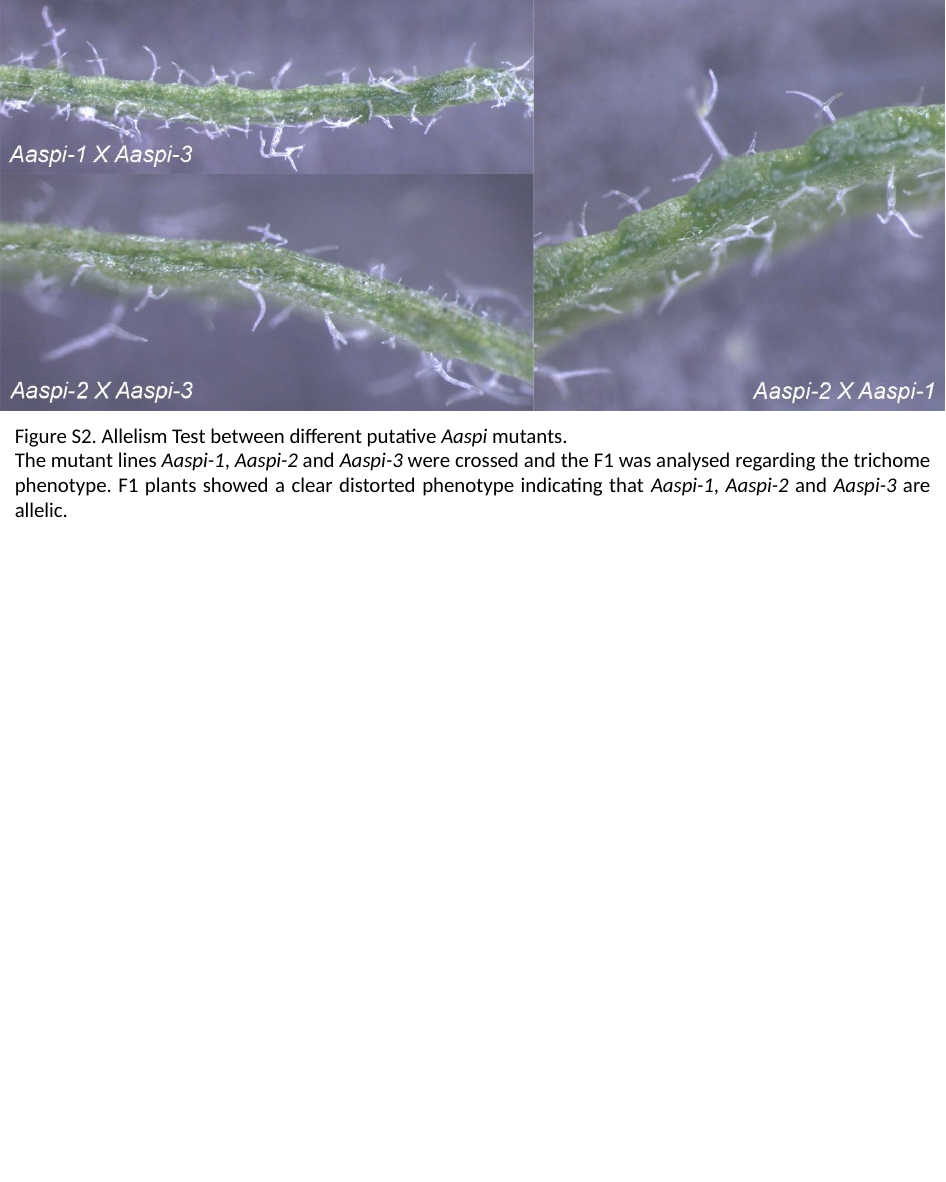

Figure S2. Allelism Test between different putative Aaspi mutants.
The mutant lines Aaspi-1, Aaspi-2 and Aaspi-3 were crossed and the F1 was analysed regarding the trichome phenotype. F1 plants showed a clear distorted phenotype indicating that Aaspi-1, Aaspi-2 and Aaspi-3 are allelic.

## Slide 3
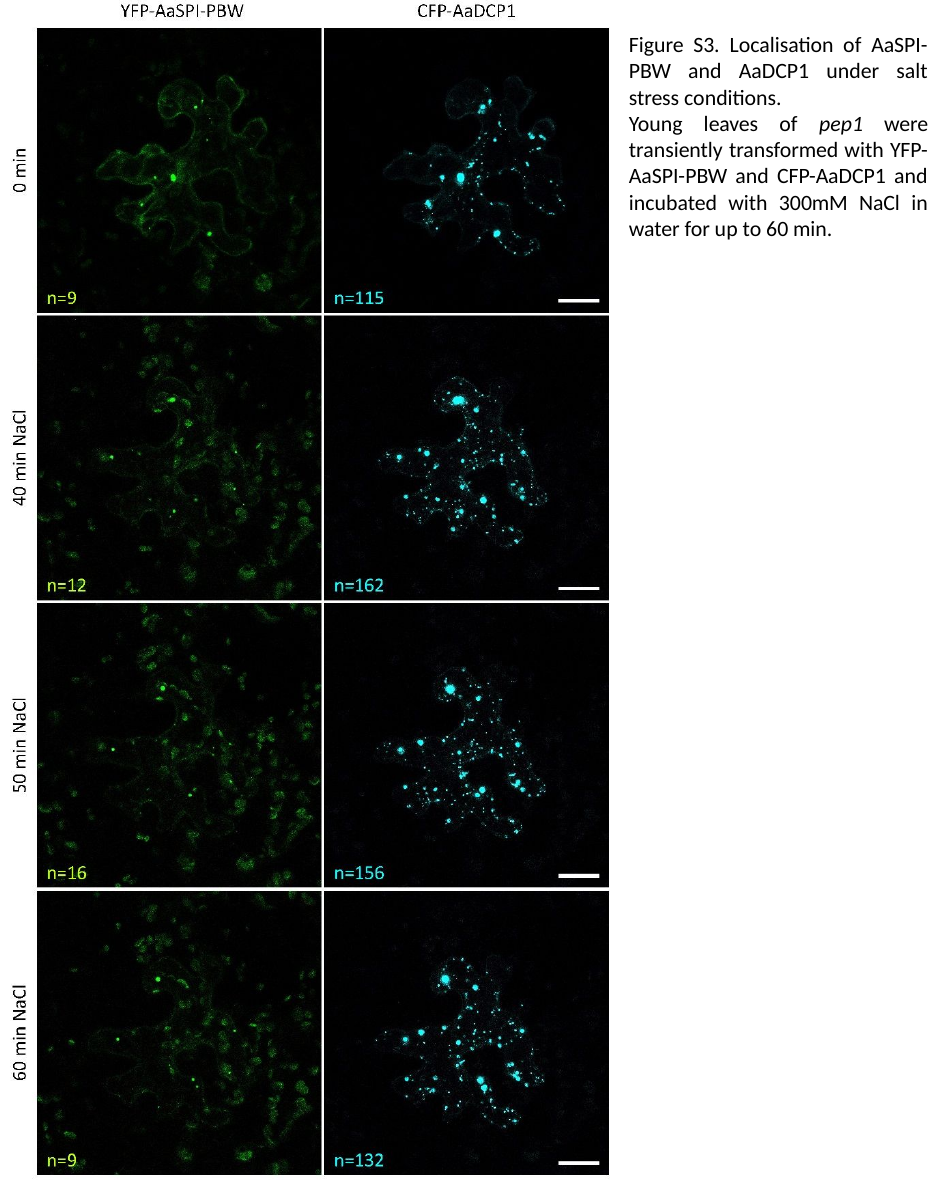

Figure S3. Localisation of AaSPI-PBW and AaDCP1 under salt stress conditions.
Young leaves of pep1 were transiently transformed with YFP-AaSPI-PBW and CFP-AaDCP1 and incubated with 300mM NaCl in water for up to 60 min.

## Slide 4
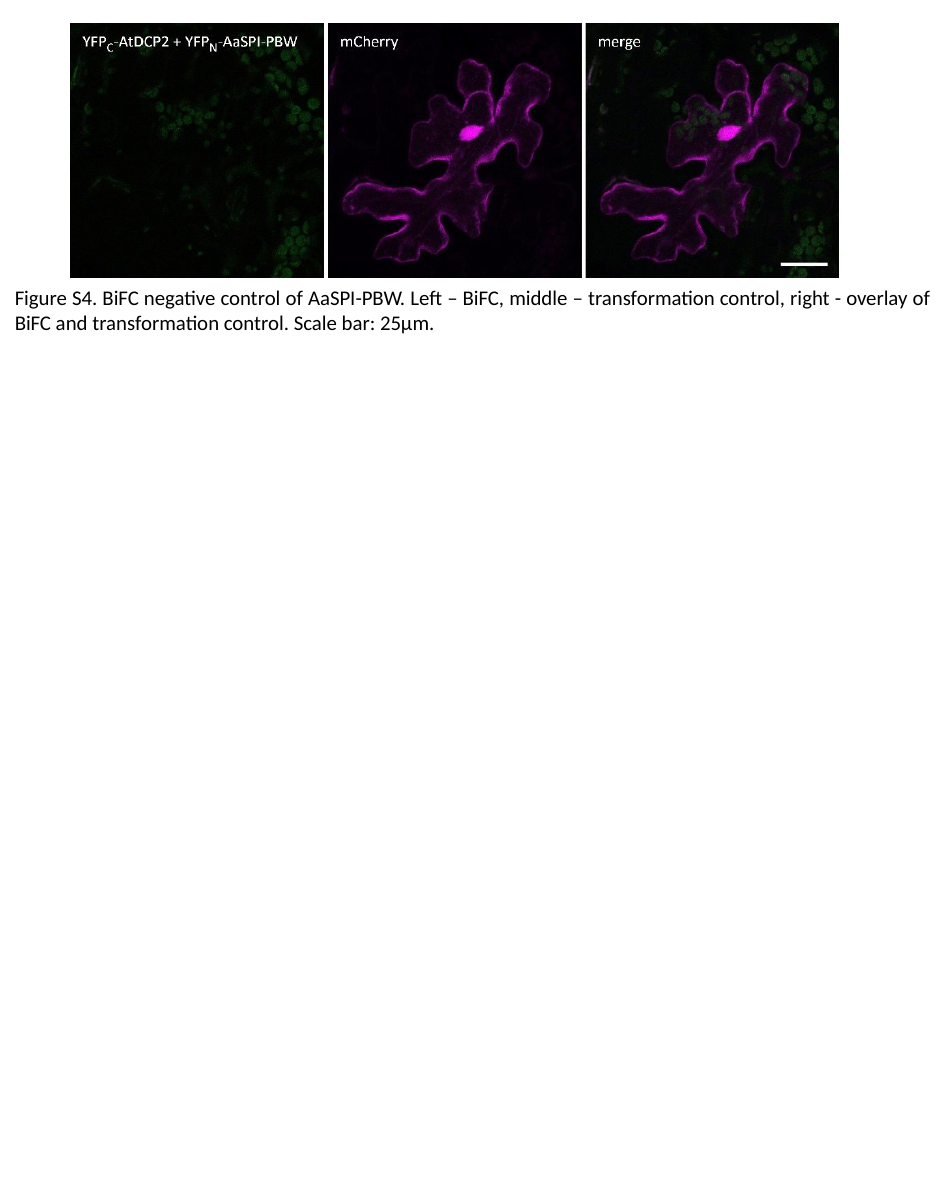

Figure S4. BiFC negative control of AaSPI-PBW. Left – BiFC, middle – transformation control, right - overlay of BiFC and transformation control. Scale bar: 25µm.

## Slide 5
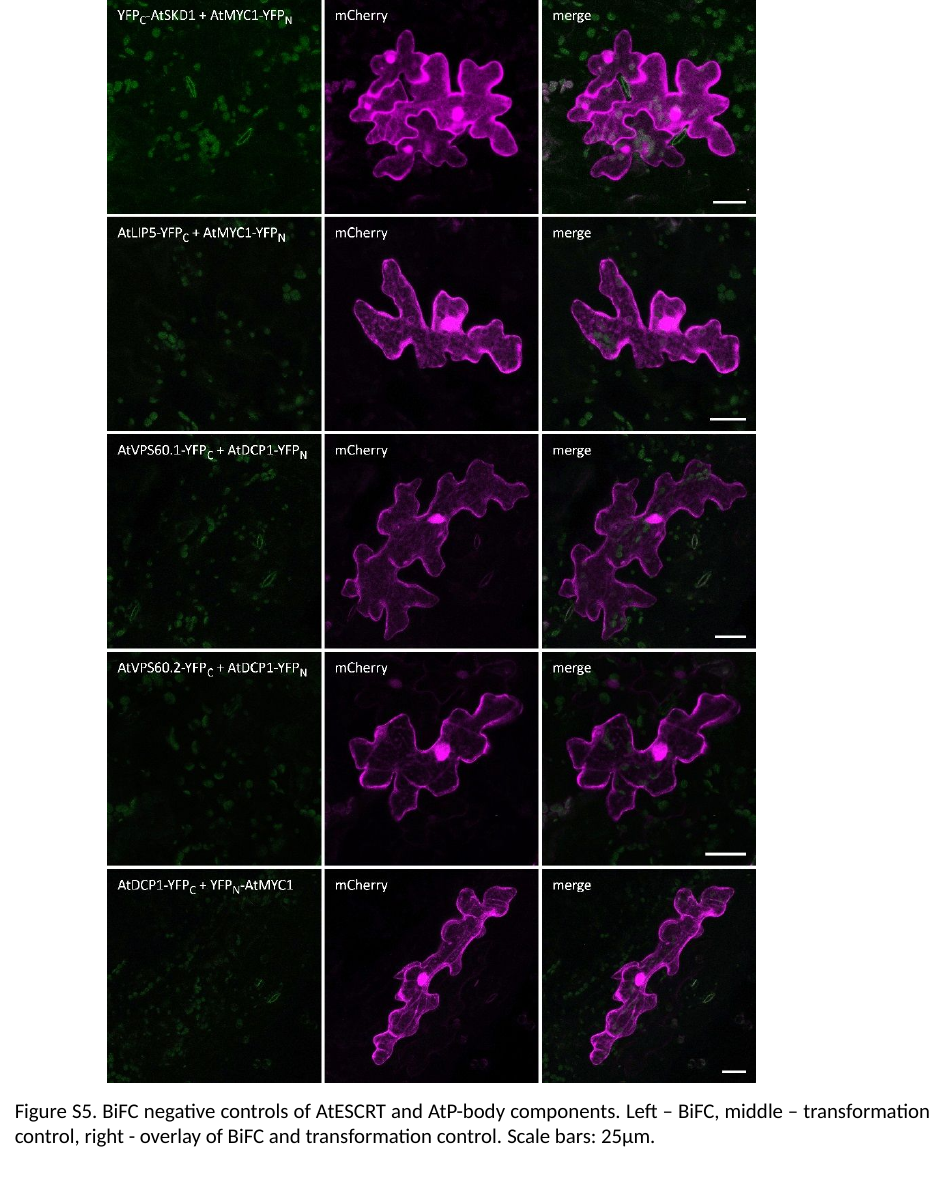

Figure S5. BiFC negative controls of AtESCRT and AtP-body components. Left – BiFC, middle – transformation control, right - overlay of BiFC and transformation control. Scale bars: 25µm.

## Slide 6
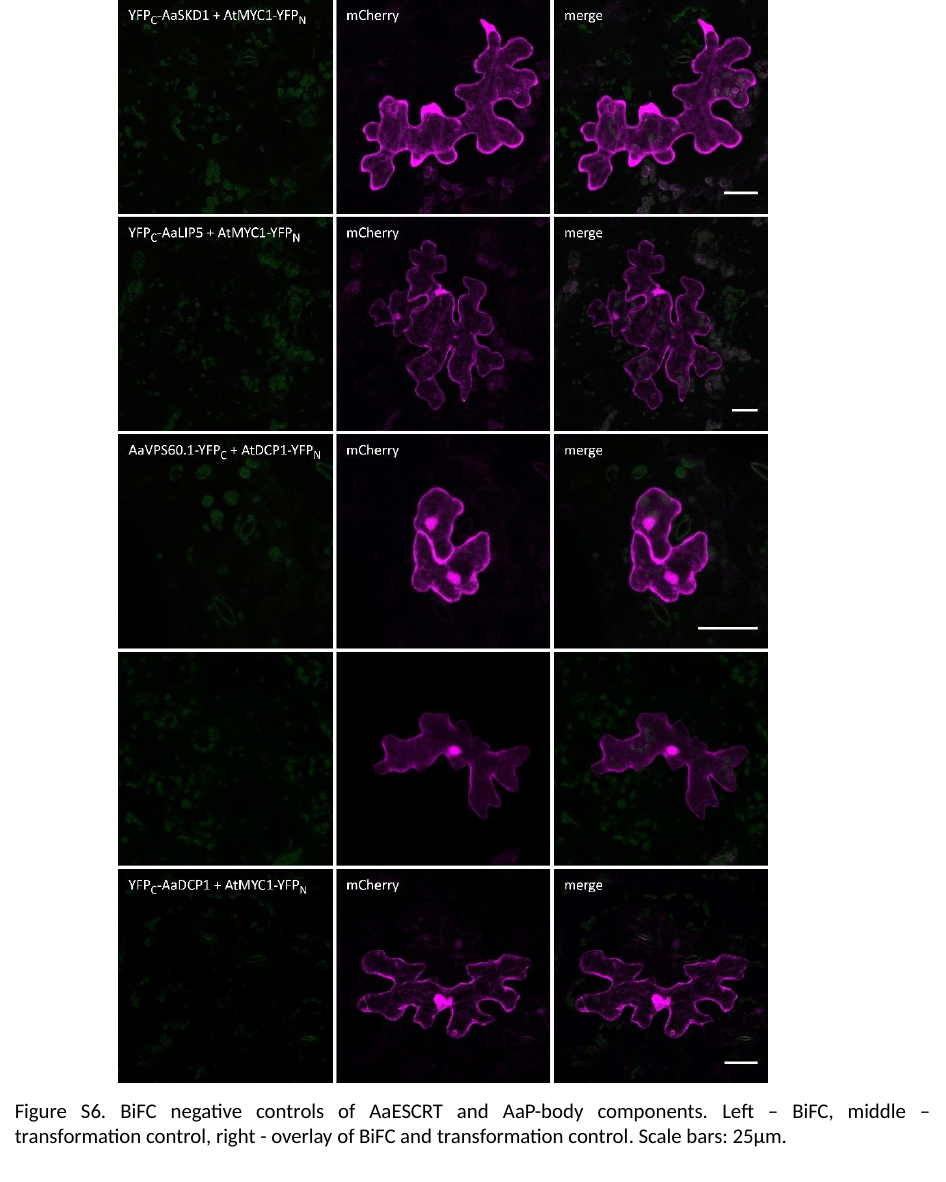

Figure S6. BiFC negative controls of AaESCRT and AaP-body components. Left – BiFC, middle – transformation control, right - overlay of BiFC and transformation control. Scale bars: 25µm.

## Slide 7
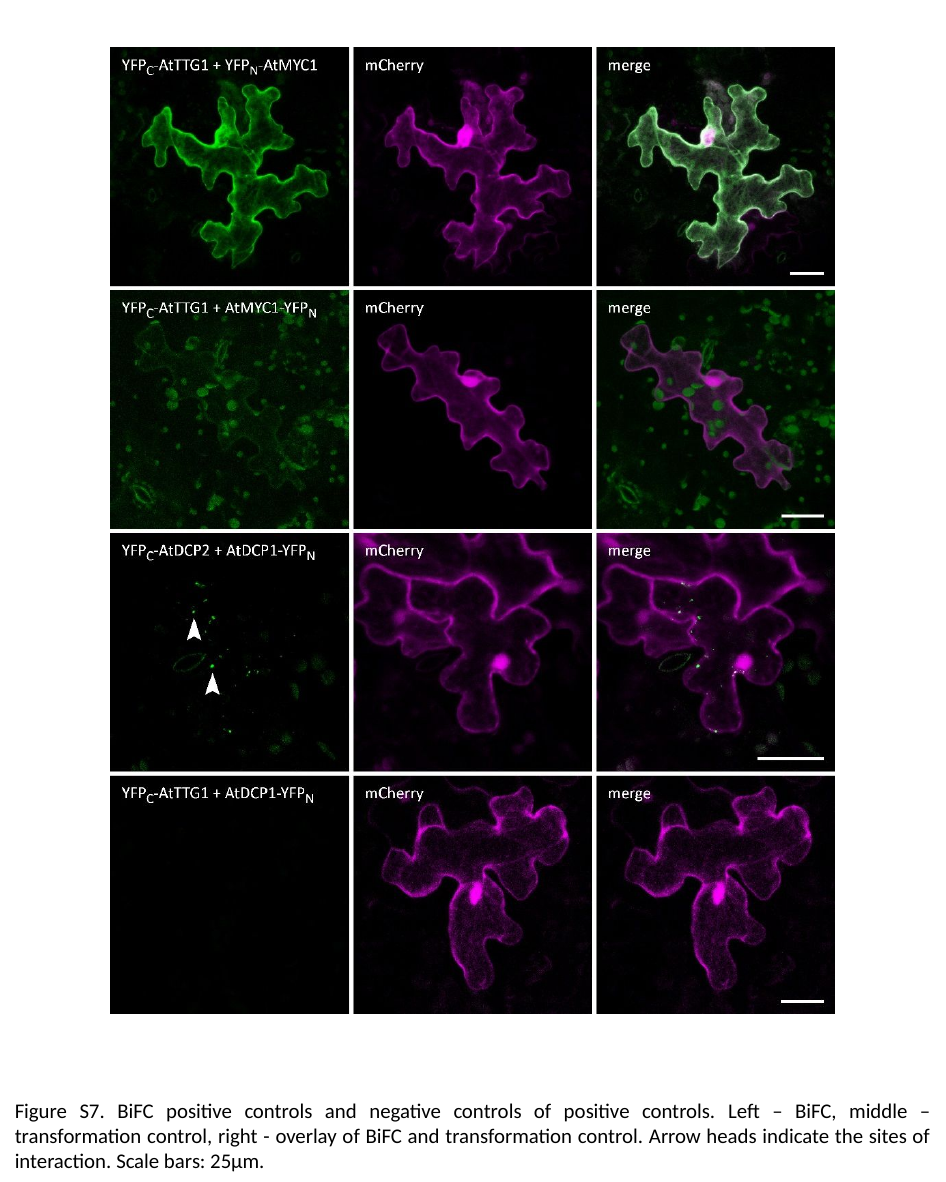

Figure S7. BiFC positive controls and negative controls of positive controls. Left – BiFC, middle – transformation control, right - overlay of BiFC and transformation control. Arrow heads indicate the sites of interaction. Scale bars: 25µm.

## Slide 8
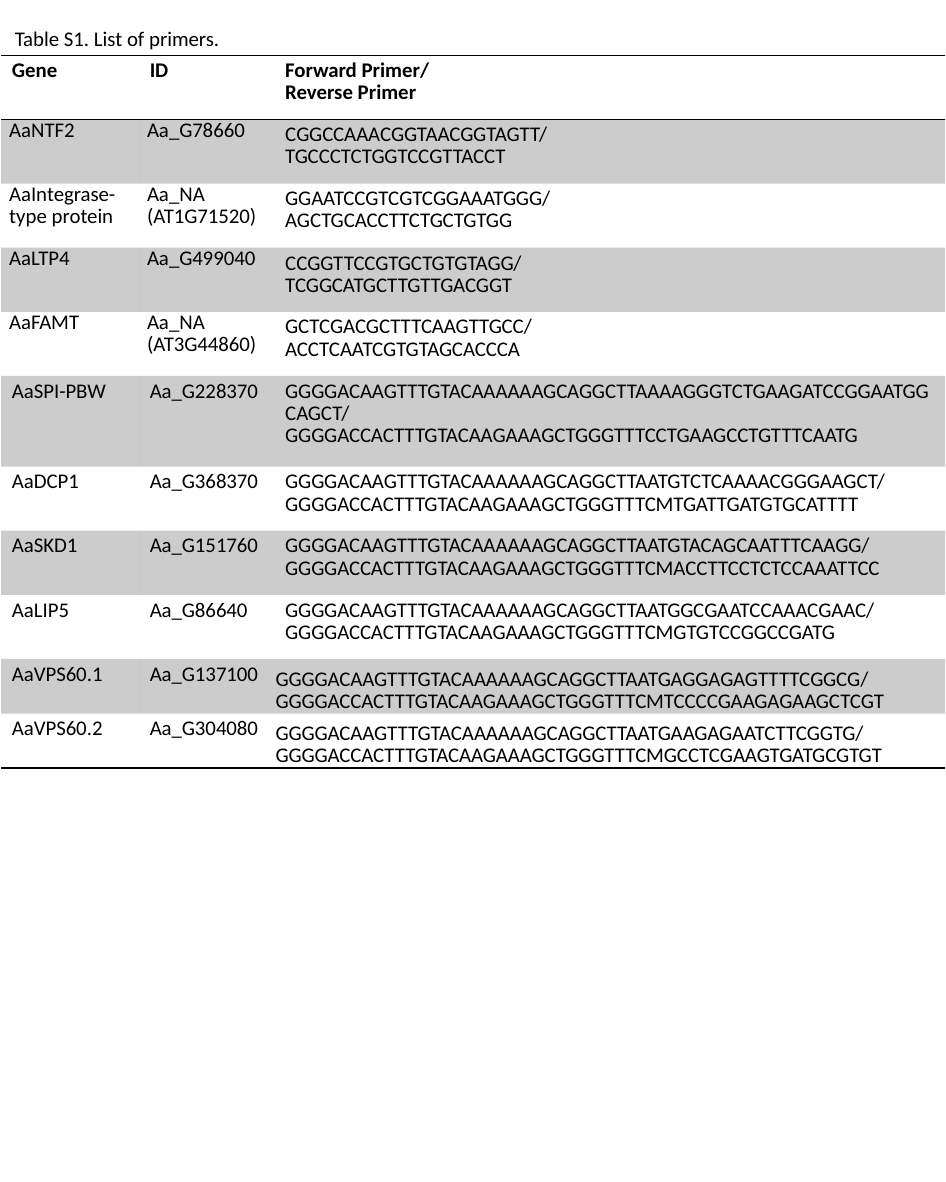

Table S1. List of primers.
| Gene | ID | Forward Primer/ Reverse Primer |
| --- | --- | --- |
| AaNTF2 | Aa\_G78660 | CGGCCAAACGGTAACGGTAGTT/ TGCCCTCTGGTCCGTTACCT |
| AaIntegrase-type protein | Aa\_NA (AT1G71520) | GGAATCCGTCGTCGGAAATGGG/ AGCTGCACCTTCTGCTGTGG |
| AaLTP4 | Aa\_G499040 | CCGGTTCCGTGCTGTGTAGG/ TCGGCATGCTTGTTGACGGT |
| AaFAMT | Aa\_NA (AT3G44860) | GCTCGACGCTTTCAAGTTGCC/ ACCTCAATCGTGTAGCACCCA |
| AaSPI-PBW | Aa\_G228370 | GGGGACAAGTTTGTACAAAAAAGCAGGCTTAAAAGGGTCTGAAGATCCGGAATGGCAGCT/ GGGGACCACTTTGTACAAGAAAGCTGGGTTTCCTGAAGCCTGTTTCAATG |
| AaDCP1 | Aa\_G368370 | GGGGACAAGTTTGTACAAAAAAGCAGGCTTAATGTCTCAAAACGGGAAGCT/ GGGGACCACTTTGTACAAGAAAGCTGGGTTTCMTGATTGATGTGCATTTT |
| AaSKD1 | Aa\_G151760 | GGGGACAAGTTTGTACAAAAAAGCAGGCTTAATGTACAGCAATTTCAAGG/ GGGGACCACTTTGTACAAGAAAGCTGGGTTTCMACCTTCCTCTCCAAATTCC |
| AaLIP5 | Aa\_G86640 | GGGGACAAGTTTGTACAAAAAAGCAGGCTTAATGGCGAATCCAAACGAAC/ GGGGACCACTTTGTACAAGAAAGCTGGGTTTCMGTGTCCGGCCGATG |
| AaVPS60.1 | Aa\_G137100 | GGGGACAAGTTTGTACAAAAAAGCAGGCTTAATGAGGAGAGTTTTCGGCG/ GGGGACCACTTTGTACAAGAAAGCTGGGTTTCMTCCCCGAAGAGAAGCTCGT |
| AaVPS60.2 | Aa\_G304080 | GGGGACAAGTTTGTACAAAAAAGCAGGCTTAATGAAGAGAATCTTCGGTG/ GGGGACCACTTTGTACAAGAAAGCTGGGTTTCMGCCTCGAAGTGATGCGTGT |

## Slide 9
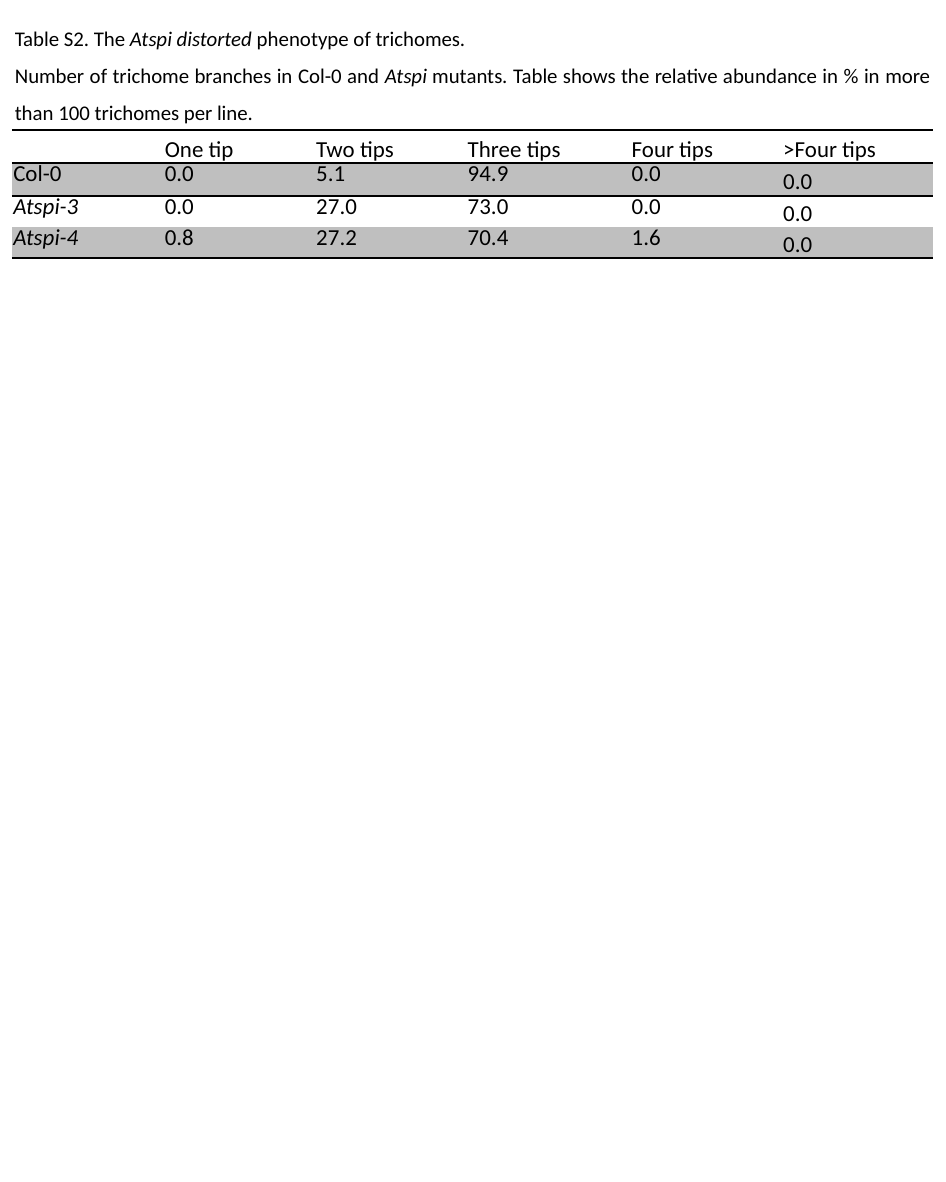

Table S2. The Atspi distorted phenotype of trichomes.
Number of trichome branches in Col-0 and Atspi mutants. Table shows the relative abundance in % in more than 100 trichomes per line.
| | One tip | Two tips | Three tips | Four tips | >Four tips |
| --- | --- | --- | --- | --- | --- |
| Col-0 | 0.0 | 5.1 | 94.9 | 0.0 | 0.0 |
| Atspi-3 | 0.0 | 27.0 | 73.0 | 0.0 | 0.0 |
| Atspi-4 | 0.8 | 27.2 | 70.4 | 1.6 | 0.0 |
